# Rapamycin-Loaded mPEG-PLGA Nanoparticles Ameliorate Hepatic Steatosis and Liver Injury in Non-alcoholic Fatty Liver Disease

**DOI:** 10.3389/fchem.2020.00407

**Published:** 2020-05-28

**Authors:** Ruifang Zhao, Meilin Zhu, Shuang Zhou, Weiyue Feng, Hanqing Chen

**Affiliations:** ^1^Department of Gastroenterology, Guangzhou Digestive Disease Center, Guangzhou First People's Hospital, School of Medicine, South China University of Technology, Guangzhou, China; ^2^National Center for Nanoscience and Technology, Beijing, China; ^3^CAS Key Laboratory for Biomedical Effects of Nanomaterials and Nanosafety, Institute of High Energy Physics, Chinese Academy of Sciences (CAS), Beijing, China; ^4^Institute of Physical Science and Information Technology, Anhui University, Hefei, China

**Keywords:** non-alcoholic fatty liver disease, mPEG-PLGA, rapamycin, hepatic steatosis, SREBP-1c, *de novo* lipogenesis, fatty acid oxidation

## Abstract

Non-alcoholic fatty liver disease (NAFLD) is characterized by excessive lipid accumulation and liver injury, and is the leading cause of chronic liver disease worldwide. There is an urgent need to develop novel pathophysiology-oriented therapy in human. Rapamycin (RAPA) has been recognized as a promising drug for alleviating hepatic steatosis on NAFLD, but the poorly water-soluble properties and side effects of RAPA limit their clinical use. In this study, we aimed to investigate the *in vitro* and *in vivo* therapeutic efficacy of biodegradable mPEG-PLGA polymers loaded with RAPA (NP-RAPA) on NAFLD. NP-RAPA were prepared by a green process using an emulsion/solvent evaporation method, the therapeutic efficacy on NAFLD were investigated on HepG2 cells incubated with oleic acid (OA) and in the livers of mice with NAFLD induced by high-fat diet (HFD). Compared with free RAPA, NP-RAPA significantly reduced lipid accumulation in HepG2 cells, and obviously ameliorated hepatic steatosis and liver injury in mice though enhancing the therapeutic efficacy of RAPA through reducing SREBP-1c-dependent *de novo* lipogenesis (DNL) and promoting PPARα-mediated fatty acid oxidation. This study suggests that mPEG-PLGA can be used as the potential therapeutic strategy and novel drug delivery for improving the efficacy of rapamycin for treatment of NAFLD.

## Introduction

Non-alcoholic fatty liver disease (NAFLD) is a prevalent hepatic manifestation of the metabolic syndrome, and encompasses a wide spectrum of chronic liver diseases, ranging from non-alcoholic fatty liver (NAFL) to non-alcoholic steatosis (NASH), liver fibrosis, cirrhosis, and ultimately hepatocellular carcinoma (HCC) (Rotman and Sanyal, 2017; Liu et al., 2020). In retrospective study, NAFLD afflicts ~30% of the adult population worldwide (Younossi et al., 2019), and its prevalence has increased about 2-fold over the past two decades (Fan, 2013). In clinical practice, NAFLD is characterized by excessive triglyceride accumulation and hepatocyte injury in the liver, which is not caused by alcohol uptake and other factors (Liu et al., 2018; Chen, 2020). However, there are no effective strategies available for NAFLD treatment. There is an urgent need to develop novel pathophysiology-oriented therapy in human NAFLD.

Recent works have revealed that sterol regulatory element-binding protein (SREBP)-1c, a critical transcription factor in regulation of *de novo* lipogenesis (DNL) and hepatic lipid accumulation, has been implicated in alcoholic and non-alcoholic steatosis (Yeh and Brunt, 2014; Chen et al., 2018). DNL, accounting for 26% of hepatic triglyceride in human subjects (Smith et al., 2020), is increased in NAFLD patients compared with healthy (Shimano and Sato, 2017), suggesting the potential therapeutic strategy through inhibition of DNL for the treatment of NAFLD. Mechanistic target of rapamycin complex 1 (mTORC1), as a central hub of nutrient sensor contributes to cellular metabolism and growth in response to physiological fluctuations of nutrients (Kim and Guan, 2019; Chen, 2020). Several studies reveal the mTORC1 signaling as an important checkpoint for SREBP-1c-mediated DNL and NAFLD pathogenesis (Lee et al., 2017; Kim and Guan, 2019). The use of specifically pharmacological inhibitor of mTOR, such as rapamycin (RAPA) as approved by the United States Food and Drug Administration (FDA) (Flaxman et al., 2019), has become the standard of care for improving fatty liver condition in alcoholic and non-alcoholic fatty liver diseases via reducing lipid synthesis or promoting lipid oxidation (Lin et al., 2013). RAPA is firstly isolated in 1975 from *Streptomyces hygroscopicus* (Othman et al., 2016), and its clinical applications for amelioration of hepatic steatosis is due to formation of the ternary complex between 12-kDa FK506-binding protein (FKBP12) and the FKBP12-rapamycin binding (FRB) (FKBP12-rapamycin-FRB) (Flaxman et al., 2019), which allosterically inhibits the kinase activity of mTORC1. RAPA administration exhibited significantly improved insulin resistance and hepatic steatosis in type 2 diabetes (T2DM) through inhibition of mTOR and alleviation of disorders of lipid metabolism (Zhou and Ye, 2018). However, the therapeutic efficacy for NAFLD is impeded by its poor solubility and low bioavailability in physiological conditions (Othman et al., 2016), and serious adverse effects have occurred due to the side effect of RAPA, which limits the clinical use (Bee et al., 2018). Therefore, more effective and safer therapy and drug delivery for RAPA in treatment of NAFLD is needed through using lower doses of RAPA to obtain the equal benefit with fewer side effects.

Rapid progress in nanotechnology in biomedicine has led to the development of the novel drug carriers for poorly water-soluble drugs to overcome their poor bioavailability and reduce their side effects (Teng et al., 2019; van der Meel et al., 2019). Polymeric nanoparticles (NPs) have been extensively reported for their prominent superiorities in potential drug delivery vehicles due to their controlled/sustained release properties, biocompatibility, and applicability to many water-insoluble drugs. Poly (D, L-lactide-co-glycolide) (PLGA) is a biodegradable polymer and is approved by FDA for biomedical applications in humans (Qureshi et al., 2016). Monomethoxy-poly(ethylene glycol) (mPEG) is the most widely used “stealth” polymer in modifying drug delivery and is classified as Generally Regarded as Safe (GRAS) by FDA (Suk et al., 2016). Now, there are more than nine FDA-approved PEGylated therapeutics in disease treatment due to their extended-circulation properties and “accelerated blood clearance (ABC)” phenomenon (Ishida et al., 2008; Weissig et al., 2014). Recently, several mPEG-PLGA copolymers, with mPEG as the hydrophilic shell and PLGA as the fabrication of bone substitute constructs, have been designed to deliver hydrophobic drugs for disease treatment (Liu et al., 2019), indicating the promising for mPEG-PLGA encapsulated with RAPA in treatment with NAFLD.

Herein, we designed and developed an efficient encapsulation of RAPA into mPEG-PLGA polymer nanoparticles (NP-RAPA) with narrow size distribution and well-dispersion stability. The NP-RAPA exerts significantly decreased triglyceride accumulation on NAFLD in HepG2 cells and in mice, compared with free RAPA. Moreover, NP-RAPA significantly improved lipid homeostasis through reducing SREBP-1c-mediated *de novo* lipogenesis and promoting PPARα-dependent fatty acid oxidation. These findings altogether demonstrated that mPEG-PLGA could be used as the new drug delivery platforms for potential therapeutic strategy in improving the efficacy of rapamycin for NAFLD treatment.

## Materials and Methods

### Chemicals and Reagents

Monomethoxy-poly(ethylene glycol) (mPEG, 5,000 Da) and poly(lactic-co-glycolic acid) (PLGA, molar ratio of D, L-lactic to glycolic acid, 75:25) were purchased from Jinan Daigang Biotechnology Co. Ltd. (Jinan, China). Rapamycin (RAPA, purity ≥99%), N,N-Diethylnicotinamide (DENA), polyvinyl alcohol (PVA), and Rhodamine B (RHO) were obtained from Sigma-Aldrich (St. Louis, MO, USA). Dulbecco's modified Eagle medium (DMEM), fetal bovine serum (FBS), penicillin, and streptomycin were obtained from by Gibco (Grand Island, NY, USA). The bicinchoninic acid (BCA) protein assay kit was obtained from Beyotime Institute of Biotechnology (Beijing, China). Liver triglyceride and cholesterol contents were determined by analytical kit from Jiancheng Bioengineering (Nanjing, China). The other chemicals and reagents with used in this study were obtained from the Sinopharm Chemical Reagent Co., Ltd. (Beijing, China) and were of analytical grade.

### Preparation and Characterization of Rapamycin-Loaded mPEG-PLGA Nanoparticles

mPEG-PLGA polymers (mPEG-PLGA) and rapamycin-loaded mPEG-PLGA nanoparticles (NP-RAPA) were prepared using an emulsion/solvent evaporation method described previously (Wang et al., 2011). In brief, 20 mg of mPEG-PLGA and 1 mg of rapamycin (RAPA) were dissolved in 1 mL of methylene chloride, and then the solution was stirred for 10 min at room temperature. After being mixed with 10 mL of 1% PVA (1% w/v), the mixture was emulsified by sonication and the solvent was evaporated by vacuum. After evaporation, the NP-RAPA was collected by centrifugation at 10,000 rpm/min for 10 min at room temperature and re-suspended in Milli-Q water for experiment.

### Characterization of Rapamycin-Loaded mPEG-PLGA Nanoparticles

The morphology and size of mPEG-PLGA polymers (mPEG-PLGA) and rapamycin-loaded mPEG-PLGA nanoparticles (NP-RAPA) were characterized by TEM (JEM-200CX, Jeol Ltd., Tokyo, Japan). The hydrodynamic diameter, zeta potential, and polydisperse index (PDI) were measured via dynamic light scattering (DLS) using a Zetasizer Nano ZS (Malvern Instrument Ltd., Worcestershire, UK).

### *In vitro* Drug Release

*In vitro* release performance of RAPA from NP-RAPA was determined using a dialysis method (Othman et al., 2016). Free rapamycin is almost insoluble in water (Chen et al., 2013). Lyophilized NP-RAPA (20–30 mg) were suspended in dissolution medium (v/v) containing 10% pure ethanol, 10% Tween-20, 25.9% N,N-Diethylnicotinamide (DENA, 3M), 10% phosphate-buffered saline (pH 7.4 and 5.5), and 44.1% Milli-Q water and transferred into the centrifuge tube (5 mL/tube) (Othman et al., 2016). To mimic the human bloodstream conditions, the tube was kept in a shaking bath at 37°C with 50 rpm. At the predetermined time intervals, the tubes were centrifuged at 15,000 rpm/min for 30 min at −4°C. The released rapamycin was quantified by high-performance liquid chromatographic (HPLC, Waters, Elstree, U.K.) method. The accumulative ratio of the released RAPA was calculated as a function of time.

### Drug Loading and Encapsulation Efficiency

Rapamycin (RAPA) was dissolved in ethanol in concentrations ranging from 0.2 to 1 mg/mL, and RAPA solution (0.2 mL) was mixed with 2 mL of mPEG-PLGA solution (2 mg/mL). Under stirring at room temperature for 2 days, the resulting NP-RAPA were collected by centrifugation at 1,000 g for 30 min. The ethanol was removed by rotary evaporation and the free RAPA was quantified by HPLC method. The drug loading capacity and encapsulation efficiency of RAPA were calculated by following formulas: Loading Content = (weight of loaded RAPA)/(total weight of NP-RAPA) × 100%, and Encapsulation Efficiency = (weight of loaded RAPA)/(weight of initially added RAPA) × 100%.

### *In vitro* Cell Model of NAFLD and Treatment

Human hepatocellular carcinoma HepG2 cells were purchased from Cell Culture Center, Institute of Basic Medical Sciences of Chinese Academy of Medical Sciences (Beijing, China) and grown in Dulbecco's modified Eagle medium (DMEM) supplemented with 10% fetal bovine serum (FBS), 100 U/mL penicillin, 100 μg/mL streptomycin, and 1% penicillin/streptomycin in a humidified atmosphere of 95% air/5% CO_2_ at 37°C. To establish the *in vitro* cell model of NAFLD, HepG2 cells were incubated with 0.4 mM oleic acid (OA, dissolved in DMEM containing 0.67% bovine serum albumin, w/v) for 24 h induce steatosis. After 24 h, the OA was withdrawn, and the cells at a density of 10^6^ cells/well were treated with mPEG-PLGA, free RAPA (0.5 μM, dissolved in 0.1% DMSO) and NP-RAPA (containing 0.5 μM RAPA) for 8 h, the cells were stained with Oil Red O to image and determine the lipid content.

### NAFLD Mouse Model and Treatment

Seven- to eight-week-old C57BL/6J male mice (22–24 g) were obtained from Beijing Vital River Experimental Animal Technology Co. Ltd (Beijing, China) and acclimatized for 1 week before the experiment. The mice were maintained in a standard environment and were fed with sterilized chow and deionized water *ad libitum* (Chen et al., 2017, 2018). All the studies were performed according to the animal protocols in compliance with laboratory animal regulations of the Ministry of Science and Technology of China and Ethics Committee of National Center for Nanoscience and Technology of China (Sep. 06, 2019, PONY-2019-FL-24).

To establish the mouse model of NAFLD, mice were fed with normal chow diet (ND, TP23302, Tophic, Nongtong, China) or high-fat diet (HFD) containing 60% calories fat (TP23302, Tophic, Nongtong, China) for 13 weeks. Free rapamycin was dosed in 4% ethanol/5% PEG 400/5% Tween-80 in water for injection solution (Chen et al., 2013). After fed with ND or HFD for 4 weeks, the mice were intravenously injected with injection solution, mPEG-PLGA, free RAPA (5 mg/kg) and NP-RAPA (mPEG-PLGA polymers containing 5 mg/kg RAPA) once a week for 8 weeks. The body weight were weighted and recorded every week.

### Histological Analysis

For histological analysis, the liver samples were collected and fixed with 10% formalin for 48 h at room temperature, and then embedded with paraffin for hematoxylin and eosin staining.

For Oil Red O study, the liver samples were embedded in OTC and cut into 7 mm thick cryosections. The cryosections were stained with Oil Red O (ORO) for steatosis levels and were counter-stained with hematoxylin for nuclei.

### Biochemistry Analysis in Plasma and Liver

For serum biochemical assess, the plasma was collected by centrifugation at 3,000 rpm/min for 10 min at 37°C. The serum biochemical parameters, including alanine aminotransferase (ALT), aspartate aminotransferase (AST), and triglyceride (TG), were determined by automatic chemistry analyzer (Celltac, MEK-6358; Nihon Kohden Co., Tokyo, Japan). For evaluation of liver function, the liver lipid was extracted following our previous protocols (Chen et al., 2018), and the content of TG was measured by commercial kits from Jiancheng Bioengineering (Nanjing, China).

### Quantitative Real-Time Polymerase Chain Reaction (qPCR) Assay

Total RNA from the frozen liver tissues (20–30 mg) was extracted by TRNzol reagent (Tiangen Biotech, Beijing, China). cDNA was reverse transcribed from the RNA (1 μg) according to the cDNA reverse transcription kit (Takara Biotechnology, Otsu, Japan) and the qPCR were performed using SYBR Green qPCR Mix kit (Life Technologies, MA, USA) on a Bio-Rad CFX Connect Real-Time PCR Detection system (Chen et al., 2018). The relative mRNA expression was normalized to internal control GAPDH. The qPCR primers used for gene expression are listed in Table 1.

**Table 1:** List of the sequences of qPCR primers used in this study.

**Genes**	**Forward primers sequences (5^**′**^-3^**′**^)**	**Reverse primers sequences (5^**′**^-3^**′**^)**
*Srebp-1c*	5**′**-GGAGCCATGGATTGCA CATT-3**′**	5**′**-GGCCCGGGAAGTC ACTGT-3**′**
*Acc1*	5**′**-TGACAGACTGATCGCAGAG AAAG-3**′**	5**′**-TGGAGAGCCCCACA CACA-3**′**
*Fasn*	5**′**-GCTGCGGAAACTTCAGG AAAT-3**′**	5**′**-AGAGACGTGTCACTCCTG GACTT-3**′**
*Scd1*	5**′**-TTCTTCTCTCACGTGG GTTG-3**′**	5**′**-CGGGCTTGTAGTACCT CCTC-3**′**
*Dgat2*	5**′**-TACTCCAAGCCCATC ACCAC-3**′**	5**′**-CAGTTCACCTCCAGCA CCTC-3**′**
*Pparα*	5**′**-GGGCAGAGCAAGTCAT CTTC-3**′**	5**′**-CCTCTGGAAGCACTGA GGAC-3**′**
*Cpt-1α*	5**′**-CCAGGCTACAGTGGGA CATT-3**′**	5**′**-GAACTTGCCCATGTCC TTGT-3**′**
*Pgc-1α*	5**′**-AAGAGCGCCGTGTGAT TTAC-3**′**	5**′**-ACGGTGCATTCCTCAA TTTC-3**′**
*Gapdh*	5**′**-TGCGACTTCAACAGCA ACTC-3**′**	5**′**-CTTGCTCAGTGTCCTT GCTG-3**′**

### Statistical Analysis

All the tests were repeated at least three times and all the data were presented as means ± standard deviations (SD). Statistical analysis was performed using GraphPad prism 6 and statistical differences were assessed using Student's *t*-test or one-way of analysis (ANOVA). *p* < 0.05 was considered as statistical significance.

## Results and Discussions

### Physicochemical Characterization of Rapamycin-Loaded mPEG-PLGA Nanoparticles

**Figure 1:**
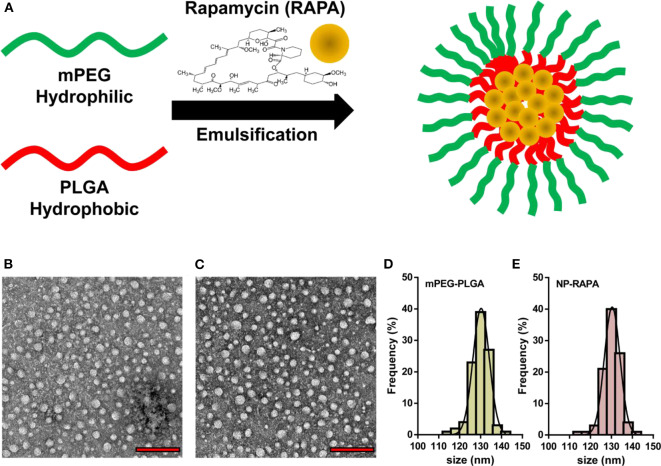
Physicochemical characterization of rapamycin-loaded mPEG-PLGA nanoparticles (NP-RAPA). **(A)** Schematic illustration of the preparation of mPEG-PLGA polymers encapsulation of rapamycin (RAPA). Representative TEM images of mPEG-PLGA **(B)** and NP-RAPA **(C)**. Scale bars, 500 nm. Size distribution of mPEG-PLGA **(D)** and NP-RAPA **(E)**.

The designed structure and preparation of mPEG-PLGA polymers and rapamycin-loaded mPEG-PLGA nanoparticles (NP-RAPA) is presented in Figure 1A, which was prepared by a green process via a water/oil/water solvent evaporation technique (Wang et al., 2011). The baseline physicochemical characteristics of the NPs are showed and listed in Figure 1 and Table 2. TEM images showed that the mPEG-PLGA and NP-RAPA yielded the monodisperse nanospheres with a smooth surface. The size distribution of the mPEG-PLGA and NP-RAPA well-dispersed in Milli-Q water had a narrow size uniformity through coulter counter analysis from TEM images, and the average size of NP-RAPA is increased from 130.4 ± 17.3 to 132.6 ± 13.5 nm, indicating the successful loading and encapsulation of RAPA on mPEG-PLGA polymer (Othman et al., 2016). The hydrodynamic diameter, zeta potential, and polydisperse index (PDI) were further measured to analyze the stability of mPEG-PLGA and NP-RAPA solution. The hydrodynamic diameter of mPEG-PLGA and NP-RAPA was 154.3 ± 14.9 to 157.5 ± 19.5 nm, and the zeta potential was −33.9 ± 0.9 to −33.7 ± 0.5 mV, respectively. The PDI was comparable between mPEG-PLGA and NP-RAPA, indicating that rapamycin-loading do not affect the stability and dispersion of mPEG-PLGA polymers.

**Table 2:** Statistical analysis of average size, hydrodynamic diameter, zeta potential, and polydispersity index (PDI) of mPEG-PLGA polymers and rapamycin-loaded mPEG-PLGA nanoparticles (NP-RAPA).

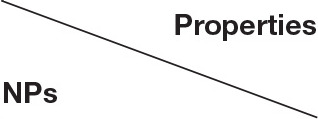	**Size (TEM)**	**Hydrodynamic dimeter**	**Zeta potential**	**PDI**
mPEG-PLGA	130.4 ± 17.3 nm	154.3 ± 14.9 nm	−33.9 ± 0.9 mV	0.166 ± 0.35
NP-RAPA	132.6 ± 13.5 nm	157.5 ± 19.5 nm	−33.7 ± 0.5 mV	0.173 ± 0.23

### Drug Loading and Release Profile of Rapamycin From NP-RAPA

**Figure 2:**
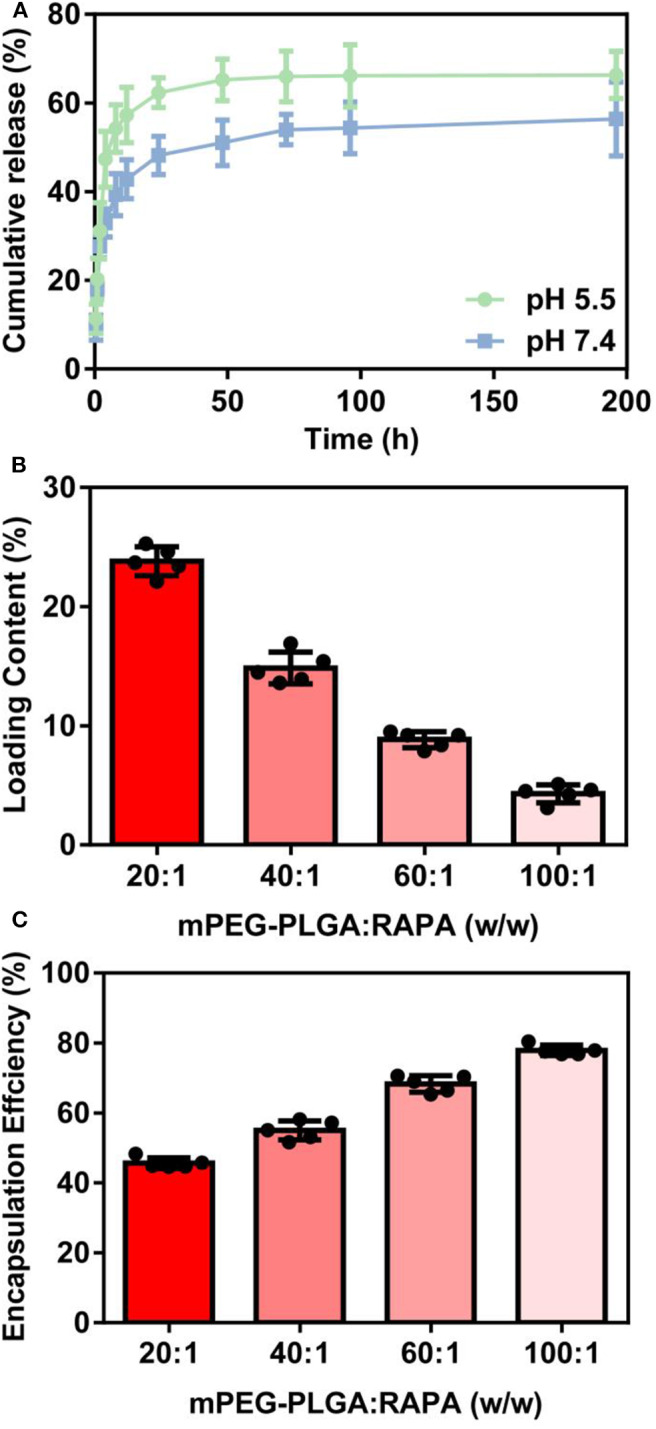
**(A)** *In vitro* drug release profiles of rapamycin (RAPA) from rapamycin-loaded mPEG-PLGA nanoparticles (NP-RAPA) at pH 5.5 and 7.4. **(B)** The loading content of RAPA in NP-RAPA. **(C)** The encapsulation efficiency of RAPA in NP-RAPA.

*In vitro* release profile can be used to determine whether dissolution of the targeted drug into carrier is enhanced by the novel drug delivery platform (Cao et al., 2018). The release curve of RAPA from NP-RAPA *in vitro* at pH 5.5 and 7.4 was shown in Figure 2A with zero time corresponding to the start of the incubation period. The NP-RAPA at pH 7.4 presented a burst release of more than 40% of RAPA in the initial 4 h. The faster release of RAPA was observed from NP-RAPA at pH 5.5 due to the simulated acidic environment of lysosome (Wang et al., 2011). After that, the release rate of RAPA from NP-RAPA was slower from 4 to 24 h (32.9–48.2% at pH 7.4, and 47.3–62.3% at pH 5.5). The release profile was approaching its plateau, and the final cumulative release of RAPA from the NP-RAPA was 56.4 and 66.3% at pH 7.4 and 5.5, respectively. For NP-RAPA, RAPA was efficiently loaded in mPEG-PLGA polymer with the highest drug loading content (LC) of 23.8% at the mPEG-PLGA/RAPA weight ratio of 20:1 (Figure 2B). The encapsulation efficiency (EE) of NP-RAPA was reduced when increasing the weight ratio of mPEG-PLGA/RAPA, and the highest EE of RAPA in mPEG-PLGA was 77.9% at the ratio of 100:1 (Figure 2C).

### Effects of NP-RAPA on *in vitro* Cell Model of NAFLD

**Figure 3:**
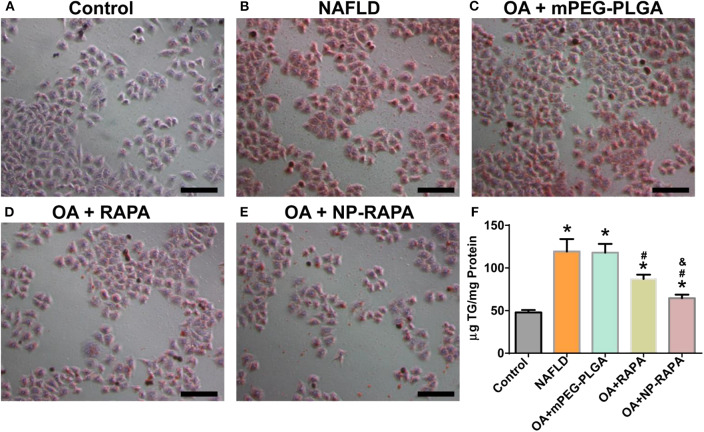
The therapeutic efficacy of biodegradable mPEG-PLGA nanoparticles loaded with rapamycin (NP-RAPA) on non-alcoholic fatty liver disease (NAFLD) in HepG2 cells. HepG2 cells were incubated with 0.4 mM OA for 24 h to induce *in vitro* cell model of NAFLD. Oil Red O staining of the cells treated with blank DMEM **(A)**, OA **(B)**, mPEG-PLGA **(C)**, free RAPA **(D)**, and NP-RAPA **(E)**. Scale bars, 50 μm. **(F)** The triglyceride content of HepG2 cells in the *in vitro* NAFLD experiment. *p < 0.05 vs. control group; ^#^*p* < 0.05 vs. OA-treated group; ^&^*p* < 0.05 vs. RAPA-treated group.

NAFLD is characterized by excessive lipid accumulation in the hepatocytes (Yu et al., 2019). In the physiological condition, intracellular lipid homeostasis is tightly controlled by nutrient sensor or signaling (Hart, 2019). Dysregulation of lipid metabolism in the liver caused by lipid accumulation leads to metabolic syndrome and contributes to the pathogenesis of NAFLD (Liangpunsakul and Chalasani, 2019). To explore the therapeutic potential of NP-RAPA on NAFLD, we first tested the effect of NP-RAPA in cellular lipid accumulation on *in vitro* cell model of NAFLD (Figure 3). HepG2 cells treated with 0.4 mM oleic acid (OA) for 24 h exhibited obvious dense ORO staining, indicating the potential of lipid accumulation and steatosis in the hepatocytes. The ORO stained lipid droplets induced by OA were significantly reduced in RAPA- and NP-RAPA-treated HepG2 cells compared with untreated and mPEG-PLGA-treated cells. More interestingly, the content of lipid accumulation was reduced to a greater extent when HepG2 cells treated with NP-RAPA than with free RAPA only. Taken together, our data indicate that NP-RAPA treatment can enhance the efficacy of RAPA on abrogation of OA-mediated lipid droplet accumulation on *in vitro* cell model of NAFLD.

### Effects of NP-RAPA on NAFLD in Mice

**Figure 4:**
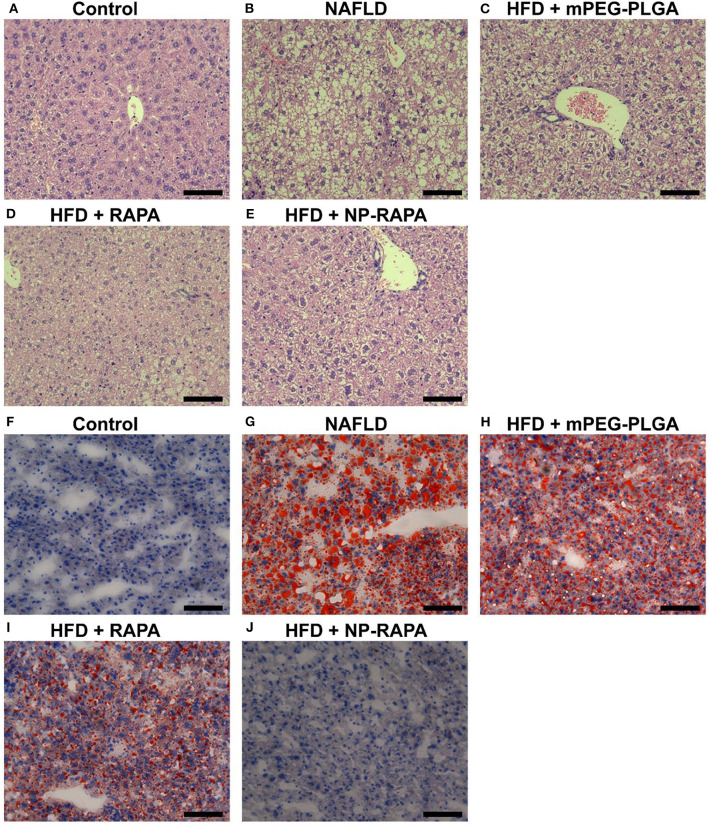
Rapamycin-loaded mPEG-PLGA nanoparticles (NP-RAPA) ameliorated HFD-induced hepatic steatosis and liver injury on NAFLD in mice. **(A–E)** Representative hematoxylin and eosin (H&E) staining of hepatic steatosis in a mouse of HFD-induced NAFLD. **(F–J)** Representative Oil Red O (ORO) staining of liver cryosections of the mice with NAFLD. Mice fed with HFD for 13 weeks together with intravenous injection of mPEG-PLGA, free RAPA and NP-RAPA. **(A,F)** NS; **(B,G)** HFD-induced NAFLD; **(C,H)** mPEG-PLGA; **(D,I)** RAPA; **(E,J)** NP-RAPA.

In order to explore the effect of NP-RAPA on NAFLD, and compare the therapeutic efficacy, mPEG-PLGA, RAPA, and NP-RAPA were intravenously injected into C57BL/6J mice during the period of HFD feeding (Figure 4). Hematoxylin and eosin (H&E) staining showed that HFD treatment resulted in lipid droplets in the hepatocytes compared with ND-treated mice, indicating the successful establishment of a mouse model of NAFLD, which has been described previously (Cao et al., 2018; Zhao et al., 2018). However, administration of mPEG-PLGA polymers did not induce the reduction in the hepatic lipid accumulation at all. RAPA only showed significantly reduced the hepatic lipid content compared with HFD group, and the NP-RAPA administration was able to more obviously ameliorate the content of hepatic lipid droplet on NAFLD, compared with free RAPA only. Results of ORO staining were also in agreement to H&E staining confirming the potential of NP-RAPA treatment to ameliorate HFD-induced NAFLD via reducing hepatic accumulation in mice.

**Figure 5:**
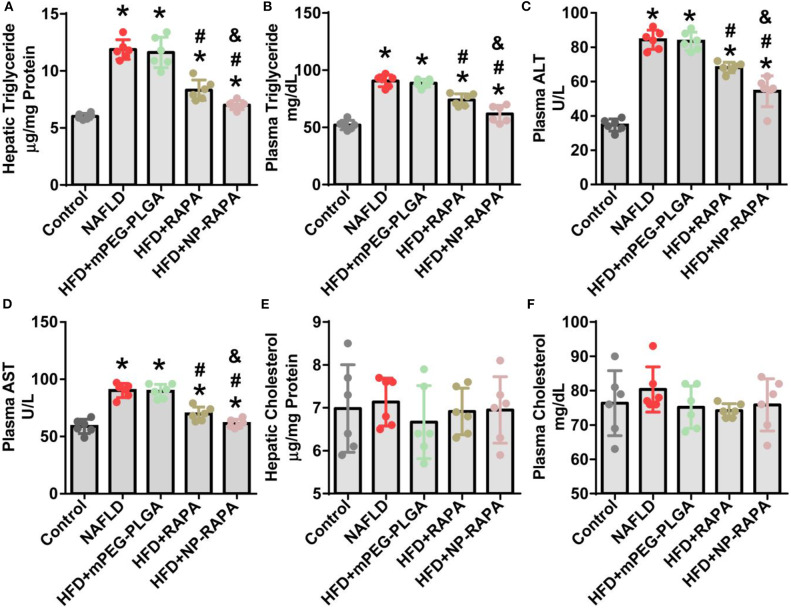
The effect of rapamycin-loaded mPEG-PLGA nanoparticles (NP-RAPA) on liver function and serum biochemical index. Compared with free RAPA, NP-RAPA administration significantly improved hepatic steatosis and ameliorated liver injury in HFD-induced NAFLD in mice. **(A)** Hepatic triglyceride; **(B)** Plasma triglyceride; **(C)** Plasma alanine aminotransferase (ALT); **(D)** Plasma aspartate aminotransferase (AST); **(E)** Hepatic cholesterol; **(F)** Plasma cholesterol. Scale bars, 100 μm. *p < 0.05 vs. control group; ^#^*p* < 0.05 vs. HFD-treated group; ^&^*p* < 0.05 vs. RAPA-treated group.

Quantitative analysis of the plasma and hepatic lipid content and liver function was shown in Figure 5. Mice treated with RAPA and NP-RAPA displayed much less hepatic triglyceride (TG) accumulation, and significantly reduced the level of serum alanine aminotransferase (ALT), aspartate aminotransferase (AST), and TG compared with HFD-induced NAFLD mice and mPEG-PLGA-treated NAFLD mice. Hepatic and plasma cholesterol levels were comparable among all the treated groups. These results altogether suggested that NP-RAPA could efficiently protect the mice from HFD-induced hepatic steatosis and liver injury in mice with NAFLD, the effects of which were better than free RAPA, confirming the potential and novel drug delivery for mPEG-PLGA in NAFLD treatment.

### NP-RAPA Reduced SREBP-1c-Mediated *de novo* Lipogenesis in NAFLD Treatment

**Figure 6:**
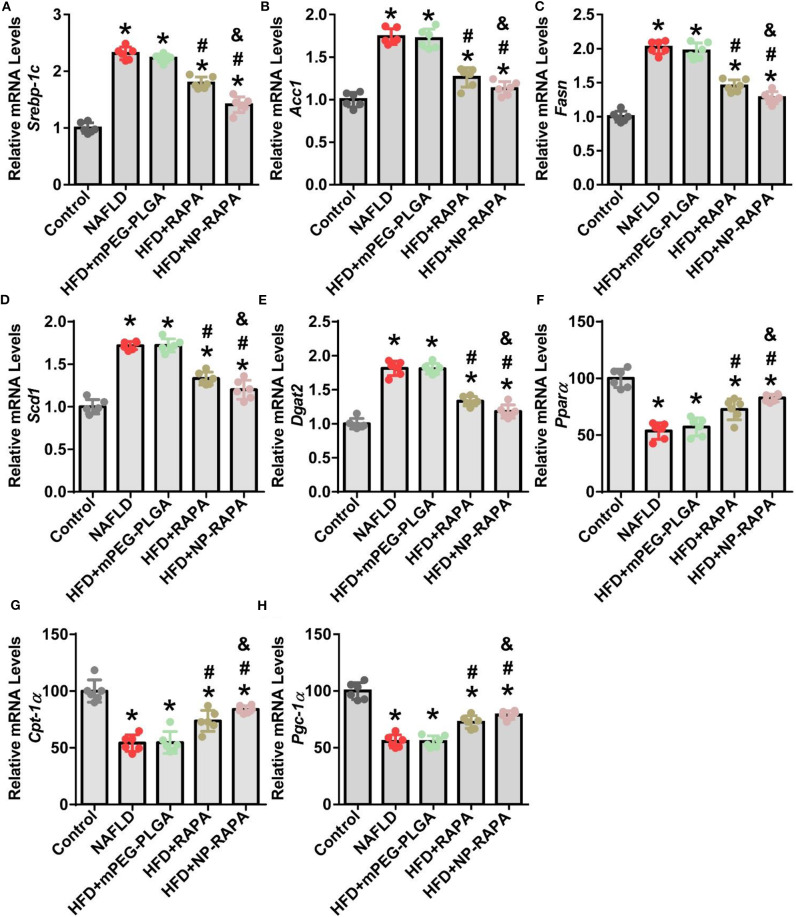
Evaluation of lipid homeostasis in the liver of HFD-induced NAFLD in mice together with NP-RAPA. Hepatic mRNA expression of SREBP-1c-mediated *de novo* lipogenesis (DNL), including *Srebp-1c* **(A)**, *Acc1* **(B)**, *Fasn* **(C)**, *Scd1* **(D)**, and *Dgat2* **(E)** in mice treated with mPEG-PLGA, free RAPA and NP-RAPA. Transcription levels of mRNAs encoding PPARα-mediated hepatic fatty acid oxidation, including *Ppara* **(F)**, *Cpt-1a* **(G)**, and *Pgc-1*α **(H)** in mice. *p < 0.05 vs. control group; ^#^*p* < 0.05 vs. HFD-treated group; ^&^*p* < 0.05 vs. RAPA-treated group.

In order to elucidate the underlying mechanisms of therapeutic efficacy of NP-RAPA in NAFLD, we determined the lipogenesis-related gene expression in the liver (Figure 6). SREBP-1c-mediated *de novo* lipogenesis (DNL) is associated with hepatic triglyceride accumulation, which contributes to hepatic steatosis and is the mainly pathological character of NAFLD (Brunt, 2010; Kawano and Cohen, 2013; Shimano and Sato, 2017; Chen et al., 2018; Chen, 2020). Consistent with an important role of RAPA as an upstream inhibitor of mTORC1 signaling in regulation of SREBP-1c-mediated *de novo* lipogenesis (DNL) (Chen et al., 2018; Chen, 2020), gene expression of lipogenesis in the liver took place. Analysis of gene expression in the liver indicated that mRNA levels of key genes involved in SREBP-1c-dependent DNL, such as *Srebp-1c* and its target genes, including *Acc1, Fasn, Scd1*, and *Dgat2* (Kawano and Cohen, 2013), were significantly increased in HFD-induced NAFLD mice. mPEG-PLGA failed to ameliorate hepatic steatosis and TG accumulation because there was no significant difference of gene expression of hepatic lipogenesis between HFD group and mPEG-PLGA group. Free RAPA significantly reduced HFD-mediated up-regulation of *Srebp-1c* and its target genes in the liver. However, NP-RAPA decreased the hepatic steatosis and triglyceride accumulation even more significantly determined by gene expression of hepatic lipogenesis. Meanwhile, HFD-induced the inhibition of peroxisome proliferator-activator receptor alpha (PPARα)-mediated fatty acid oxidation was recovered in RAPA treatment, especially in NP-RAPA group, as evidenced by increased expression of genes *Ppar*α, *Cpt-1*α, and *Pgc-1*α (Brocker et al., 2018; Chen et al., 2018). Thus, NP-RAPA protects against HFD-induced hepatic steatosis and liver injury at least in part through enhancing the role of the therapeutic efficacy of RAPA in down-regulation of SREBP-1c-mediated *de novo* lipogenesis and up-regulation of PPARα-dependent fatty acid oxidation in NAFLD.

## Conclusions

NAFLD is the leading cause of chronic liver disease worldwide, and there are no effective strategies available for NAFLD treatment currently (Eshraghian, 2017; Issa et al., 2018; Younossi et al., 2019). In this study, we successfully designed and developed a novel drug delivery using biodegradable mPEG-PLGA polymers for enhancing the therapeutic efficacy of rapamycin in treatment of NAFLD *in vitro* and *in vivo*. RAPA-loaded mPEG-PLGA nanoparticles (NP-RAPA) were prepared using an emulsion/solvent evaporation method and its therapeutic efficacy on NAFLD was investigated in HepG2 cells and in mice. Compared to free RAPA, NP-RAPA significantly decreased lipid content in HepG2 cells treated with OA and ameliorated hepatic steatosis and liver injury in mice with NAFLD induced by high-fat diet (HFD). The molecular mechanism of the inhibitory effects of NP-RAPA on NALFD is due to reduce REBP-1c-dependent *de novo* lipogenesis (DNL) and promote PPARα-mediated fatty acid oxidation in the liver of mice. This study suggests that mPEG-PLGA can be used as the potential therapeutic strategy and novel drug delivery for improving the efficacy of rapamycin for treatment of NAFLD.

## Data Availability Statement

The raw data supporting the conclusions of this article will be made available by the authors, without undue reservation, to any qualified researcher.

## Ethics Statement

The animal study was reviewed and approved by the Ethics Committee of the National Center for Nanoscience and Technology of China.

## Author Contributions

The project was conceptually designed by HC. The majority of the experiments were performed by RZ and MZ, assisted SZ and WF. Data analysis and interpretation were carried out by RZ and HC. The manuscript was prepared by HC. All authors discussed the results and implications and commented on the manuscript.

## Conflict of Interest

The authors declare that the research was conducted in the absence of any commercial or financial relationships that could be construed as a potential conflict of interest.
